# Precision hybrid technique for direct SAPIEN-3 ultra™ implantation in extensive mitral annular calcification

**DOI:** 10.3389/fmed.2026.1748471

**Published:** 2026-05-21

**Authors:** Gianluca Lucchese, Rajdeep Bilkhu, Antonio Bivona, Vincenzo Caruso

**Affiliations:** 1 Cardiovascular Department, St. Thomas’ Hospital, London, United Kingdom; 2 School of Biomedical Engineering and Imaging Sciences, King’s College London, London, United Kingdom

**Keywords:** hybrid procedure, MAC, mitral valve, TAVI, transcatheter valves

## Abstract

Mitral valve surgery in patients with extensive mitral annular calcification remains challenging. Different surgical approaches have been described: from a complete decalcification and reconstruction of the mitral annulus to other techniques where a full debridement was avoided to minimize the risk of possible peri-operative complications. In the current era of transcatheter valve implantation, the issue of mitral annulus calcification has been addressed by some with the adoption of a hybrid technique, where a transcatheter aortic valve is implanted in mitral position, during open heart surgery. This technique is emerging as a treatment option for high-risk patients although it is still an off-label procedure and needs further standardization. We describe here our surgical technique to implant a SAPIEN-3 Ultra™ in mitral position, addressing some of the key points in the surgical technique: the appropriate sizing and the phase of deployment of the prosthesis.

## 1 Introduction

Extensive mitral annular calcification (MAC) is the result of a chronic degenerative process of the mitral annulus and represents a formidable challenge during mitral valve (MV) surgery (1). It can influence the surgical management, also affecting the outcome with an increased risk of potentially fatal complications such as left ventricular rupture, intractable hemorrhages, atrioventricular disruption and ischaemia due to disturbances of the circumflex coronary artery (2).

The preferred and more classical surgical approach of complete decalcification followed by reconstruction of the mitral annulus and then valve repair or replacement, has been investigated through different studies (3–11) (Table 1).

**Table 1:** Study on mitral annulus calcification (MAC) treatment.

Study	Time	Patients (mean age years)	Mean F-U (months)	Technique	Complications	Mortality	FrReop (years)	Long-term survival (years)
A. “Resect”—debridement of calcified tissue								
Carpentier AF (3) (1996)	1986–1994	68 (62)	40	En bloc resection, annular reconstruction with atrial muscle flap, MVr or MVR	None	2.9%	87% (6)	93% (6)
Feindel CM (4) (2003)	1985–2000	54 (63)	49	En bloc resection, annular reconstruction with bovine pericardium, MVr or MVR	RTT (*n* = 5), PPM (*n* = 11)	9.3%	89% (4)	65% (7)
D’Alessandro C (5) (2007)	1995–2005	124 (66)	50	En bloc resection, leaflet sliding plasty, MVr with ring annuloplasty, or MVR	PPM (*n* = 3) Stroke (*n* = 7), LCx graft (*n* = 2)	14%	Not stated	76% (4)
Uchimuro T (6) (2016)	2004–2013	61 (70)	38	En bloc resection, annular reconstruction, then MVr or MVR	LV pseudoaneurysm (*n* = 1), RO (*n* = 1), stroke (*n* = 2)	6.6%	98.3% (4)	75.6% (4)
Tomsic Al (7) (2019)	2002–2015	75 (70)	56	En bloc resection, annular reconstruction, MVr or MVR.	Early repair failure (*n* = 8), stroke (*n* = 2), R (*n* = 3)	4%	98.3% (7)	78.7% (7)
B. “Respect”—avoidance of annular decalcification								
Hussain ST (8) (2013)	2006–2011	20 (75)	8	Anterior leaflet transposed posteriorly, minimal calcium debridement, PTFE felt washer inserted	RTT (*n* = 1), stroke (*n* = 2)	5%	–	50% (4)
Salhiyyah K (9) (2017)	2001–2011	61 (75)	40	Mitral annulus preserved, removal of large protruding pieces of calcium, MVR	Death (*n* = 1). Stroke (*n* = 1). RTT (*n* = 3, 6.7%), CHB (*n* = 7, 16%).	6.7%	100% (1)	78.8% (4)
El Sabbagh A (10) (2018)	2014–2016	6 (81)	1	Left atriotomy (*n* = 4), vertical trans-septal (*n* = 2) deployment of balloon expandable THV*	No LVOT; Valve migration (*n* = 1), Severe PVL (*n* = 4) Death (*n* = 3)	50%	–	No long-term follow-up
Russell HM (11) (2018)	2017–2018	8 (75)	1	Left atriotomy, PTFE felt strip, AMVL excised, Sapien 3 THV** deployed	No major complications	0%	–	No long-term follow-up

F-U, follow-up; FrReop, freedom from re-operation; AMVL, anterior mitral valve leaflet; CHB, complete heart block, LCx, left circumflex coronary artery; LV, left ventricle; LVOTO, left ventricular outflow obstruction; MAC, mitral annular calcification; mmHg, millimeter of mercury; MR, mitral regurgitation; MV, mitral valve; MVR, mitral valve replacement; MVr, mitral valve repair; PPM, permanent pacemaker; PVL, paravalvular leakage; PTFE, polytetrafluoroethylene; RO, re-operation; RTT, return to theatre for bleeding; THV, transcatheter heart valve. *CUSA, Cavitron Surgical Systems Inc., Stanford, CT, USA. **Edwards Sapien 3 transcatheter aortic valve, Edwards Lifesciences, Irvine, California. A, resect and debridement technique; B, respect technique.

Alternatively, conservative and less radical approaches have been described (12–14), which although safer, are characterized by a relatively high risk of prosthesis dehiscence and severe paravalvular leak (PVL) requiring multiple interventions.

The hybrid technique with a transcatheter aortic valve is implanted in mitral position (surgical valve in MAC: SViMAC), during open heart surgery, presents multiple advantages (Table 2) (15–17). An extensive annular decalcification will not be necessary, which will reduce surgical risk; the technique will also be easily reproducible in traditional sternotomy and in minimally invasive approaches. Additionally, the chance of removing the anterior mitral leaflet (AML), during open-heart surgery, significantly decreases the risk of left ventricle outflow tract (LVOT) obstruction. This approach contrasts with interventional transcatheter mitral valve implantation (TMVR) in MAC, as it addresses LVOT obstruction, considered one of the main anatomical limitations of the technique.

**Table 2:** Hybrid technique: international registries.

Outcome	TMVR in MAC Global Registry (11) (*n* = 116)	STS/ACC Registry (12) (*n* = 100)	TMVR Registry (13) (*n* = 58)
First ViMAC enrolment	2012	2013	2015
Location	International	US	International
Centres	51	49	40
Trans-septal	40.5%	43%	53.4%
Edwards Sapien	98.3%	100%	81%
Warfarin post-procedure	66.7%	N/A	N/A
30-day outcomes			
All-cause death	25.0%	21.8%	34.5%
Cardiovascular death	13.0%	12.0%	N/A
Stroke	4.3%	6.3%	3.9%
Valve embolism	4.3%	1.6%	6.9%
MV re-intervention	7.7%	6.3%	13.8%
LVOT obstruction	11.2%~	10.0%^#^	39.7%*
Hemolytic anaemia	3.4%	N/A	N/A
MR grade >mild	15.5%	5.7%	13.2%
1-year outcomes			
All-cause death	53.7%	N/A	62.8%
Cardiovascular death	23.5%	N/A	N/A
Stroke	6.6%	N/A	N/A

ACC, American college of cardiology; LVOT, left ventricular outflow obstruction; MAC, mitral annular calcification; MR, mitral regurgitation; MV, mitral valve; STS, society of thoracic surgeons; TMVR, transcatheter mitral valve replacement; ViMAC, Valve-in-mitral annular calcification.

While the use of transcatheter prostheses in open MV surgery is still considered off-label and requires further standardization, we have developed a systematic surgical procedure that appears effective and easily reproducible. Aim of this study is to describe our technique of implant; the initial results seem promising, with satisfactory early and mid-term outcomes.

## 2 Materials and methods

### 2.1 Definition and cohort

All patients underwent pre-operative trans-thoracic echocardiogram (TTE). Severe MAC was defined following recent collaborative position statement (18) as presence of echo dense structure at the junction of the atrioventricular groove and posterior mitral leaflet > 270°. A Euro Score II (ES II) > 5 was utilized to identify high-risk patients. After surgery, TTE was used to assess the severity of PVL, using a 3-class grading scheme (mild, moderate, severe) (19).

### 2.2 Study design

This was a prospective cohort study, initiated with the aim to demonstrate our surgical technique of SViMAC. Primary outcome was all-cause operative mortality (defined as in-hospital mortality or 30-day mortality, including patients who died after 30 days but without discharge). Secondary outcomes were mortality at follow-up and post-operative comorbidities stroke [persistent (more than 24 h) neurological deficits and confirmation of brain infarction or hemorrhage on imaging], acute kidney injury (decline in renal function with need for dialysis), respiratory failure (reintubation, tracheostomy or need of prolonged ventilation), re-exploration [return to the theatre for postoperative bleeding and/or pericardial effusion or cardiac arrest, deep sternal wound infection(wound infection occurring within 30 days from surgery)].

### 2.3 Statistical analysis

Continuous data were expressed as medians and interquartile ranges (IQR); categorical variables were reported as counts (*n*) and percentages (%). Due the small sample size, only a descriptive statistical analysis was performed.

### 2.4 Equipment

Our SViMAC technique involves the use of the SAPIEN-3 Ultra™ valve. This valve has a balloon-expandable cobalt chromium alloy frame that offers high radial strength for circularity and optimal hemodynamics, along with a low frame height and open cell geometry. It also features an outer polyethylene terephthalate skirt to minimize PVL. These attributes, along with the low profile of the prosthesis, make this valve particularly well-suited for surgical implantation in the mitral position.

### 2.5 Step-to-step surgical implantation

The surgical approach can be either *via* a median sternotomy or a mini right anterior thoracotomy, as the reduced annular decalcification and consequently surgical manipulation is ideal for minimally invasive surgery.

Bicaval cannulation is performed to institute cardio-pulmonary by-pass (CPB). The heart is arrested and protected with antegrade, cold and hematic cardioplegia. The left atrium (LA) is entered through the Sondergaard’s groove, and the mitral valve is exposed and carefully inspected.

Firstly, the AML is removed. This step, together with the deployment position, are determinant to reduce the risk of LVOT obstruction. The advantage of the open technique over the totally interventional procedure, is essentially due to the removal of the AML which, in presence of a small left ventricle (LV) and/or an acute aorto-mitral angle, may lead to a significant sub-aortic obstruction.

**Figure 1:**
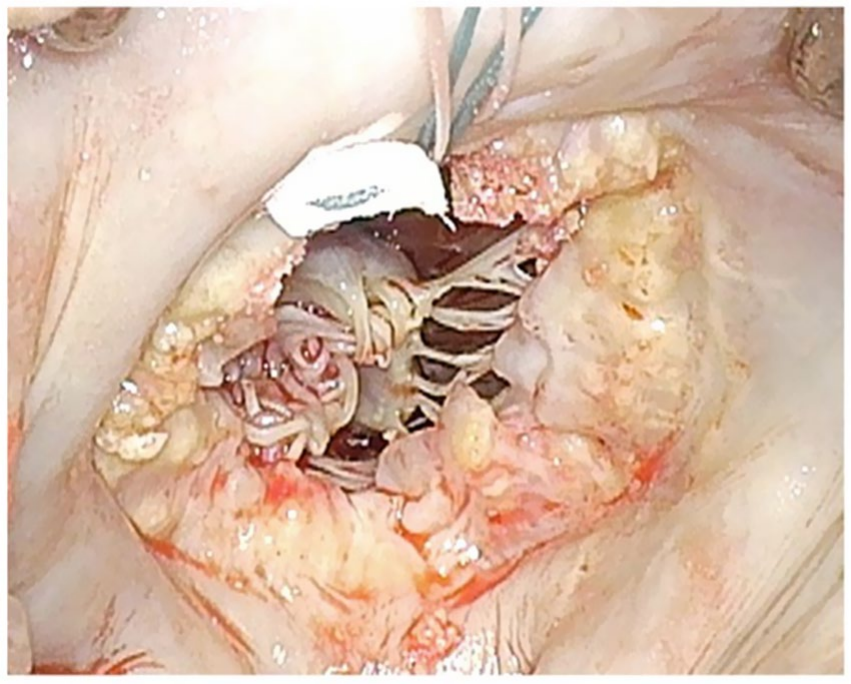
Extensive calcification of the mitral annulus is present, with significant calcification of the posterior mitral leaflet in the forefront. The anterior mitral leaflet has already been excised. A 2–0 Ethibond Excel® Polyester suture has been placed through the left atrial wall at the postero-medial commissure.

A few initial horizontal mattress sutures using 2–0 Ethibond Excel® Polyester, re-inforced with pledgets, are placed through the left atrial wall, starting from the anterior mitral annulus and continuing circumferentially, 1 cm above the mitral annulus (Figure 1).

**Figure 2:**
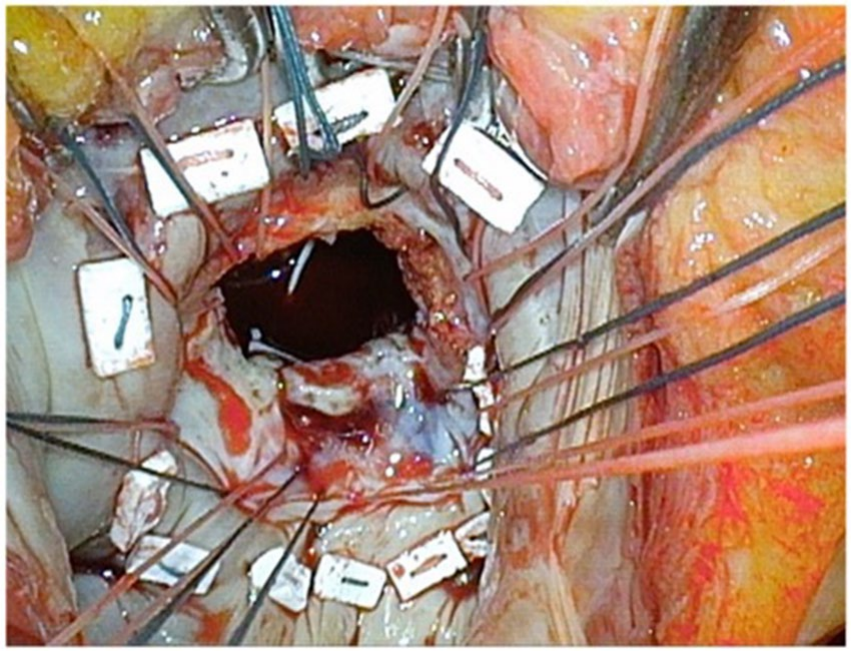
Superficial decalcification of the posterior mitral leaflet in order to allow a relatively large opening to accommodate a bigger prosthesis. Also, a smoother residual surface of the posterior mitral leaflet contributes to reduce the risk of residual paravalvular leak.

A superficial posterior mitral leaflet (PML) debridement is also performed at this stage with the aim to increase the geometric valve area and create a smoother and regular surface of the PML to reduce the risk of PVL post-deployment (Figure 2).

Additionally, we mark the two mitral commissures with a sterile pen to ensure proper orientation of the SAPIEN-3 Ultra™ during deployment. This allows us to align the commissures of the SAPIEN 3 prosthesis with the mitral commissures, keeping it away from the aortic valve and minimizing the risk of obstruction in the LVOT.

The valve sizing is a key step of the procedure. The experience of the operator with TAVI is fundamental to guide the decision making. Generally, considering the relatively large size of a mitral valve, the decision is to be taken between a 26 mm and a 29 mm SAPIEN-3 Ultra™ to avoid patient prosthesis mismatch (PPM) and provide good grip post-deployment.

We use a fully inflated 25 mm balloon to size the mitral valve. If this fits tightly, a 26 mm valve will be chosen whereas a 29 mm will be preferred if the balloon fits loosely inside the annulus.

**Figure 3:**
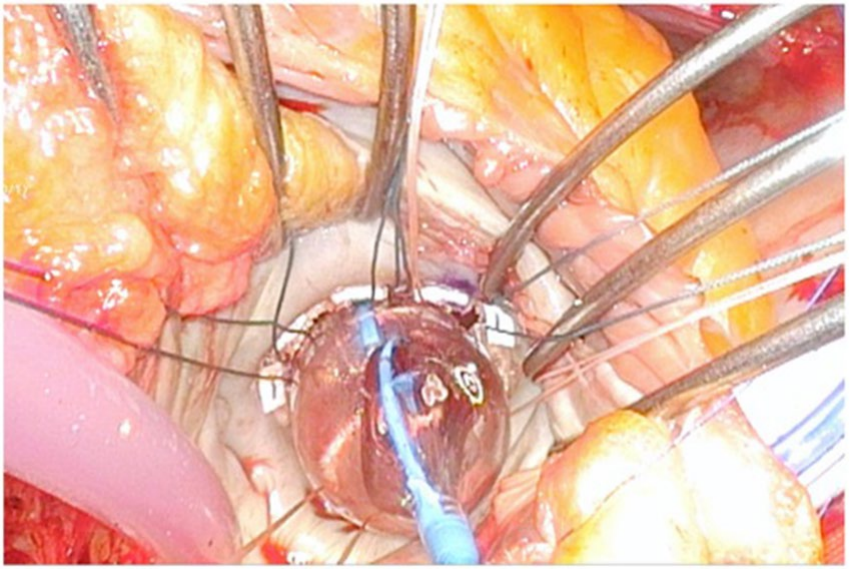
A 25 mm balloon fits tightly into the annulus, therefore we have opted for a 26 mm valve. However, we have overfilled the balloon with 2 mL for a safer anchoring and to compensate the lack of calcium of the anterior mitral annulus.

It’s important to note that the SAPIEN-3 Ultra™ can be over-filled or under-filled by up to 5 mL to achieve a better fit in the annulus. An adjustment of 2 mL results in a 1 mm increase in diameter. Also, we recommend to carefully take into consideration the LV size and the possible risk of LVOT obstruction when opting for a definitive size, keeping in mind that a larger diameter corresponds to a higher prosthesis. Thus, a 29 mm valve would be preferable in bigger LV (end-diastolic diameter > 5.5 cm) whereas a 26 mm one should be considered in smaller LV (end-diastolic diameter < 4 cm), perhaps overfilled when required (Figure 3).

The SAPIEN-3 Ultra™ is then mounted onto the deployment system making sure that its skirt is facing the LA, upside-down compared to the usual fashion for trans-femoral TAVI.

The valve is carefully positioned at the level of the mitral annulus and orthogonal to the native valvular plane. The initial inflation of the balloon needs to be performed gradually to ensure that the correct position of the SAPIEN-3 Ultra™ is maintained.

The presence of the outer skirt allows deployment up to 5-8 mm above the valvular plane without requiring a handcrafted 10 mm Teflon felt skirt sutured along the outer side the prosthetic valve, as previously described (20).

**Figure 4:**
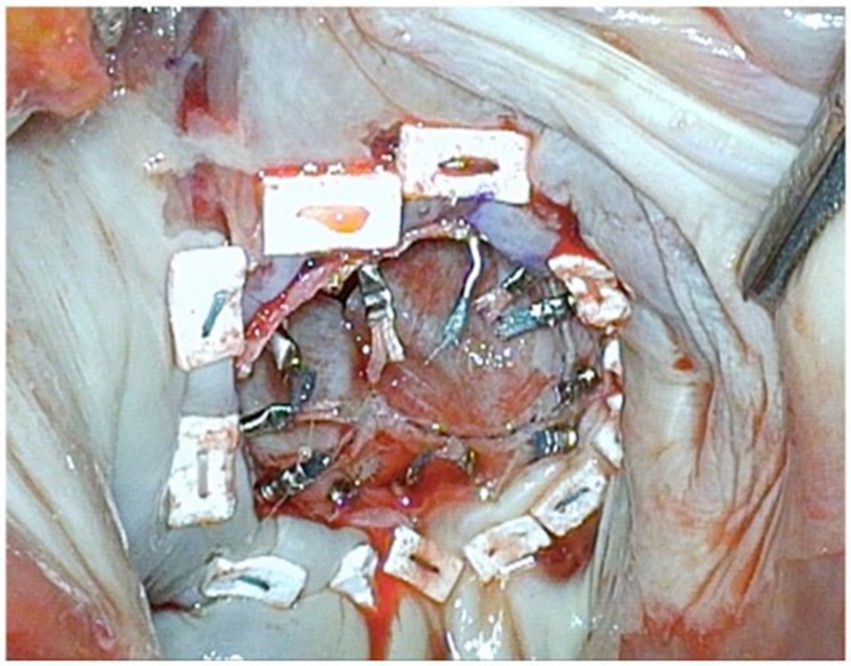
The final deployment is orthogonal to the valvular plane and a few mm into the left atrium to reduce the risk of left ventricle outflow tract obstruction. The mitral annulus is still the level of outer skirt of the SAPIEN-3 Ultra™. The stitches are carefully passed through the SAPIEN-3 Ultra™ (in the usual fashion of conventional valve implantation) directed from inside to outside the valve skirt; the suture line at the base of the leaflets can be used as reference. In the picture, the head of the papillary muscles has been resected. This is useful in case of heavily calcified papillary muscles and small left ventricle.

Deploying the prosthesis higher into the LA, further reduces the risk of LVOT obstruction, but we advise against a deployment over the height of the outer skirt to minimize the risk of PVL. The pledgetted sutures are finally passed through the outer skirt of the valve completing the implantation (Figure 4). The stitches must be carefully passed through the SAPIEN-3 Ultra™ making sure not to catch the valve leaflets—the suture line at the base of the leaflets can be used as reference.

Finally, a hydraulic saline test is performed to verify the correct opening and closing of the SAPIEN-3 Ultra™ and detect any obvious leak. Closure of the left atriotomy is completed in usual fashion. A transesophageal echocardiogram needs to be performed post-CPB to check the correct position and function of the prosthesis and exclude significant PVL and/or LVOT obstruction.

## 3 Results

In our initial experience, we performed SViMAC in seven patients (median age: 69 years, interquartile range (IQR):60, 83) with severe MAC and high-surgical risk, utilizing median sternotomy (*n* = 6, 86%) and right anterior thoracotomy (*n* = 1, 14%). All, except one, patients were female sex (86%). Median ES II was 6.2, IQR: 5, 28. Other baseline characteristics are shown in Table 3.

**Table 3:** Baseline characteristics.

	Full cohort (*n* = 7)	
Age (years) (median IQR)	69	60, 83
Female sex, *n* (%)	6	86
BMI (median, IQR)	39	31, 42
Diabetes, *n* (%)	4	57
Hypertension, *n* (%)	7	100
NYHA class		
III	1	14
IV	6	86
Chronic lung disease, *n* (%)	5	71
Redo surgery, *n* (%)	2	29
LVEF (%) (median, IQR)	50	35, 60
Euroscore II (median, IQR)	6.2	5, 28

NYHA, New York heart association; LVEF, left ventricular ejection fraction.

Associated procedures were performed in five patients (71%, coronary artery by-pass graft, *n* = 2, 40%, aortic valve replacement, *n* = 3, 60%). A size 26 mm TAVI valve was used in all cases (*n* = 6, 86) except one (*n* = 1, 14%; valve size 29 mm) (Table 4).

**Table 4:** Intra-operative and post-operative characteristics.

	Full cohort (*n* = 7)	
TAVI in MAC alone, *n* (%)	2	29
Associated procedure^*^, *n* (%)	5	71
TAVI size		
26 mm	6	86
29 mm	1	14
Surgical approach		
Full sternotomy, n (%)	6	86
RAT, n (%)	1	14
CPB minutes (median, IQR)	138	120, 211
XCT minutes (median, IQR)	112	100, 190
Intra-operative mortality, *n* (%)	0	0
Follow-up mortality, *n* (%)	2	29
Stroke, *n* (%)	0	0
Acute kidney injury, *n* (%)	3	43
Respiratory failure, *n* (%)	3	43
Re-exploration, *n* (%)	1	14
Paravalvular leak		
None, *n* (%)	3	43
Mild, *n* (%)	4	57
Mitral mean gradient (mmHg), (median, IQR)	4.5	3, 7

TAVI, Trans aortic valve implantation; MAC, mitral annulus calcification; RAT, right anterior thoracotomy; CPB, cardiopulmonary bypass; XCT, cross-clamp time.

There was no intraoperative mortality (*n* = 0, 0%) and excellent early and mid-term postoperative outcomes, with no stroke events, none or mild PVL in all cases and a median of mean mitral valve gradient of 4.5 mmHg, IQR: 3, 7. One patient required re-exploration for late pericardial effusion. At median follow-up of 22.5 months (interquartile range (IQR): 10–18), mortality was relatively high (*n* = 2, 29%) and occurred for those undergoing SViMAC and concomitant procedures.

## 4 Discussion—conclusion

Our described surgical implantation of a transcatheter SAPIEN-3 Ultra™, performed either through a full sternotomy or a minimally invasive approach, is a reproducible and effective technique that can be executed in a standardized manner. We adopted this strategy in seven patients, achieving no intraoperative mortality (*n* = 0, 0%) and excellent early and mid-term postoperative outcomes However, it’s important to recognize that high mortality during follow-up as mitral MAC represents only the tip of a more extensive cardiovascular disease.

Our results are consistent with and extend the existing literature demonstrating the use of SViMAC in severe MAC (21, 22). In a recent study (23), SViMAC showed mild PVL (4.2%) acceptable valve hemodynamics; also, the frequency of post-operative events was low while mortality was > 35%, suggesting that in this cohort may be driven by patients’ underlying comorbidities as opposed to the procedure itself. In the Surgical Implantation of Transcatheter Valve in Native Mitral Annular Calcification (SITRAL) (ClinicalTrials.gov Identifier: NCT02830204) study is now completed but awaiting results (24).

Severe MAC remains a challenging condition during mitral valve surgery. Characteristics associated with MAC include increased age, female gender, high body mass index and previous smoking history (25). It has also been found independently associated with increased incidence of cardiovascular disease, chronic kidney disease and death (26, 27).

The surgical debridement techniques are characterized by three main drawbacks. Firstly, the MAC can be rarely removed *en-bloc*; secondarily, the decalcification may produce damage to the endocardium with increased structural fragility that could compromise also the consistency of the myocardial tissue to hold the patch in place. Finally, these operations are technically challenging and long and might lead to serious postoperative complications in the elderly and high-risk patients.

Our described surgical implantation of a transcatheter SAPIEN-3 Ultra™, performed either through a full sternotomy or a minimally invasive approach, is a reproducible and effective technique that can be executed in a standardized manner. The primary advantages of our technique include the avoidance of extensive calcium debridement and the elimination of the need for annular sutures when implanting the valve. These factors, along with a standardized sizing method, make the procedure easy to perform in various settings, including endoscopic surgery. The SAPIEN-3 Ultra™ enables the safe use of a relatively larger prosthesis in cases of MAC, which is often linked to a small mitral annulus and a potential risk of patient-prosthesis mismatch (PPM). Nevertheless, with this technique there are no extra costs compared to the classical surgical approach, except from the cost of the SAPIEN-3 Ultra™ valve itself (28).

The only concern remains the durability of the transcatheter prostheses in mitral position, but again, these considerations need to be contextualized to the outcome of a subgroup of patients already very high-risk for mid-term cardiovascular mortality.

### 4.1 Limitations

Several limitations merit consideration. First, this is an observational analysis with a small sample size and therefore subject to residual confounding. Second, the analysis reflects practice patterns within a single center and may not be fully generalizable to other centers without similar experience or equipment.

## Data Availability

The raw data supporting the conclusions of this article will be made available by the authors, without undue reservation.
